# Effect of Temperature Range and Kilning Time on the Occurrence of Polycyclic Aromatic Hydrocarbons in Malt

**DOI:** 10.3390/foods12030454

**Published:** 2023-01-18

**Authors:** Kristina Habschied, Brankica Kartalović, Dragan Kovačević, Vinko Krstanović, Krešimir Mastanjević

**Affiliations:** 1 Faculty of Food Technology Osijek, Josip Juraj Strossmayer University of Osijek, F. Kuhača 20, 31000 Osijek, Croatia; 2 Scientific Veterinary Institute Novi Sad, Rumenački put 20, 21000 Novi Sad, Serbia

**Keywords:** PAHs, malt, kilning

## Abstract

Kilning is an integral part of malt production; it ensures grain and enzyme preservation. Kilning temperatures can range between 80 and 220 °C, depending on the type of malt that is being produced. Polycyclic aromatic hydrocarbons (PAHs) are prone to appear at higher temperatures and are generally designated as undesirable in food and beverages. Sixteen PAHs are framed in legislation, but there is a lack of scientific data related to PAHs in malt, malt-related foods (bread, cookies) and beverages (whisky, malted non-alcoholic beverages). The aim of this paper was to assess and quantify the occurrence of different PAHs in malts exposed to different kilning temperatures (50–210°) over a variable time frame. The results indicate that some of the PAHs detected at lower temperatures disappear when malt is exposed to high temperatures (>100 °C). Phenanthrene was no longer detected at 100 °C and indeno [1,2,3-cd] pyrene at 130 °C, while fluorene, anthracene and benzo (a) anthracene were not quantified at 170 °C. The results of this research can be implemented in food safety legislation since foods available to children utilize malted flour (bread, cookies, bakery goods, etc.) due to its enzymatic activity or as a colour additive.

## 1. Introduction

Malt is a basic raw material in beer and whisky production [1,2]. Malt flour is commonly used as an additive for various breads and baking products as a colour enhancer or for its enzymatic activity. Kilning temperatures can reach 220 °C and this could result in an increased concentration of polycyclic aromatic hydrocarbons (PAHs) in beer, whisky and bread and bakery products [3,4]. These commodities are consumed worldwide and are available to a large number of people. Moreover, bakery products are available to children as well [5] and, therefore, it is important to monitor and assess the intake of PAHs through different foods. All these products can contain different amounts of PAHs that originate from malt.

PAHs are a result of pyrolytic processes that involve incomplete combustion of wood, organic matter, coal or oil, forest fires, volcanic eruptions and carbonization [5,6,7]. According to Wenzl et al. [8], PAHs can contain two–four fused aromatic rings and are known as lower-molecular-weight PAHs (LMW-PAHs) while PAHs with a higher number of fused aromatic rings are known as higher-molecular-weight PAHs (HMW-PAHs). They are both lipophilic and their solubility decreases with increasing molecular weight [9]. As a result of incomplete combustion of fossil fuels [10], PAHs present a serious health hazard to humans [11,12]. Even though PAHs were originally designated as environmental pollutants, they are commonly found in foodstuff, especially in foods exposed to smoking, frying, cooking and grilling [13,14,15,16,17,18,19,20,21]. Charbroiling and smoking foods significantly increases PAH levels in food and beverages [10]. PAHs show various degrees of carcinogenicity but, in general, they all contribute to the carcinogenicity of other PAHs [4,16].

PAHs can be found in different foods that contain fat molecules [13,18]. However, multiple factors affect PAH concentration in foods: smoking method, wood type, duration of smoking and, of course, type of food [15]. To decrease the possibility of PAH concentrations in foods, Codex Alimentarius [22] established several recommendations for smoking exposure. The scientific literature that deals with PAHs in cereals is scarce. In research conducted so far, PAHs have been identified in breakfast cereals and bread (maximum values for bread were 0.22 µg kg^−1^ and 0.87 µg kg^−1^ for cereals) [23]. Maize grain samples dried at air temperatures of 60 °C, 60/80 °C and 80 °C also contained PAHs [24]. Chrysene was found in all dried samples in concentrations above 6 µg kg^−1^ (at 60 °C concentration was 6.18 µg kg^−1^ and at 80 °C was 7.02 μg kg^−1^). Corn grains that were not subjected to drying contained chrysene. Rice grains [25] and coffee beans [26] are also exposed to drying and they showed different results. For example, PAHs in rice grains depend on the source of heating, and the highest total PAH concentration was found when drying with wood (131.6 µg kg^−1^). Interestingly, there was a reduction in certain PAHs in coffee beans after roasting [27]. The sum of PAHs ranged from 0.015 to 0.105 mgL^−1^ (*Coffea arabica* brews) and 0.011 to 0.111 mg/L (*Coffea canephora* brews) [26]. A study by Rascón et al. [28] correlated the process of drying with levels of PAHs in granola, chocolate granola and milk-filled cereals. The results showed that three breakfast cereal foodstuffs had higher levels of PAH4 (3.1–7.5 µg kg^−1^) than prescribed by the European commission [29]. They [30] investigated PAHs in beers as well and reported that beers contain naphthalene (340–1500 ng L^−1^) and anthracene (320–2200 ng L^−1^). 

PAH molecules have different degrees of carcinogenic properties, but they all contribute to the general carcinogenic properties of certain foods exposed to smoking or other sources of PAHs. Thus, the European Food Safety Authority (EFSA) established the concentrations of benzo[a]pyrene (BaP) and the sum of the concentrations of four PAHs (benzo[a]pyrene (BaP), benz[a]anthracene (BaA), benzo [b] fluoranthene (BbF) and chrysene (Chry) (PAH4) [31]) as main the indicators of PAH toxicity via foods. According to European Commission (EU) regulations [29], the maximum allowable concentration of BaP in processed cereal-based foods is 1 μg kg^−1^, and the ∑PAH4 concentrations are set to 1 μg kg^−1^ as well. However, not many papers describe PAH presence in beer [21,32,33] and malt [4,34,35]. This research is an extension of a preliminary survey the authors conducted on commercially available malts [4]. The preliminary research showed that some of the analysed commercially available malts contained high concentrations of BaA, reaching over 737 µg/kg (black malt), while the lowest level was quantified in amber malt (60.53 µg/kg). Pilsner and cara-120 malts had high values for BaA −134.26 µg/kg and 210.7 µg/kg. This study was set to investigate the temperatures at which certain PAHs develop and assess whether kilning duration affects the concentration of PAHs in malts. 

## 2. Materials and Methods

Barley sample was purchased from the Agricultural Institute Osijek (50 kg).

### 2.1. Micromalting Procedure

Micromalting was performed according to MEBAK^®^ [36]. Thus, 250 g of barley was soaked in tap water (500 mL), according to the procedure described in MEBAK^®^ [35] and shown in Table 1. Soaking and germination were performed in a climate chamber (Euclid, d.o.o, Vinkovci, Croatia). In total, 15 batches were malted, resulting in cca 4 kg of malt. The kilning of green malt was adjusted according to the basic MEBAK^®^ protocol with rising temperatures every hour from 50 to 210 °C and the last two samples were subjected to high temperatures (190 °C and 210 °C) for two hours (as shown in Table 1). Kilning was performed in a drying oven Memmert UFE 400 (Memmert GmbH, Schwabach, Germany). After kilning, malt was cooled down to room temperature and then transferred into paper bags (Eko-Kart, Zagreb, Croatia) and kept at room temperature for two weeks for moisture equilibration. Moisture was determined after 14 days according to MEBAK^®^ standard procedure and was 4%. After two weeks, the samples were milled on IKA^®^ M20 mill (IKA^®^-Werke GmbH & Co. KG, Staufen, Germany) to powder and then sealed in glass jars (125 mL) with glass caps (Eisco Scientific LLC, Victor, NY, USA). To prevent external contamination of samples, jars and caps were rinsed with acetone, marked and forwarded for analysis.

### 2.2. PAH Analysis

For the preparation of PAH standards, a 16PAH mix (Ultra Scientific, North Kingstown, RI, USA) was used (500 ± 0.2 µg/mL). According to the Association of Analytical Communities’ (AOAC) official method 2007.01 for extraction and clean up, the samples were prepared as described by [37] using multiresidue preparations (QuEChERS). GC-MS parameters were adjusted, as described by Mastanjević et al. [15] as well.

The PAH determination method was modified, according to the accredited method ISO 17025. Validation of precision, reproducibility, accuracy, linearity, LOQ (limit of quantification), LOD (limit of detection) and uncertainty are shown in Table S1. To minimize and reduce or completely eliminate the interference with the malt matrix, a calibration using a blank sample was carried out. A detailed description of the analysis is described in a preliminary research paper published by [2]. Table 2 contains a list of PAHs analysed in this research.

### 2.3. Statistical Analysis

Analysis of variance (ANOVA) and Fisher’s least significant difference test (LSD) were conducted, and the least statistical significance was set to be *p* < 0.05. Statistica 13.1. (TIBCO Software Inc., Palo Alto, CA, USA) was the software of choice for this data set.

## 3. Results and Discussion

Barley seeds are exposed to combustion products from fossil fuels through mechanization from the time of sowing to harvest and can contain certain amounts of PAHs. Before malting, to avoid an overestimation of the PAH content that could be formed due to the chemical reactions during the kilning, we provided an analysis of a barley sample, as can be seen in Table 3. Chrysene and the sum of ∑PAH4 in barley exceed the EU-regulation-prescribed limit of 1 μgkg^−1^ and amount to 1.118 μgkg^−1^ (chrysene) and 1.391 μg kg^−1^ (∑PAH4).

Malt sample 1, exposed to 50 °C for 16 h (according to the standard kilning procedure described in [36]) showed an increased value for chrysene as well, 1.452 μg kg^−1^. ∑PAH4 was 1.704 μg kg^−1^ for this sample. Sample 2, malt exposed to 60 °C for 1 h, showed an increase in ∑PAH4 to 1.843 μg kg^−1^. This increase is mostly the result of the rise in the chrysene level to 1.542 μg kg^−1^. This increasing level trend continues for all samples and almost all PAHs. However, phenanthrene can no longer be detected at 100 °C in sample 6. At 130 °C, indeno 123 cd pyrene is also no longer detectable in malt sample 9. At 170 °C, more PAHs (fluorene, anthracene) are not detectable, and at 190 °C, acenaphthene disappears as well. They presumably react with some of the food ingredients and mask their form into an undetectable/unknown compound. ∑PAH4 reached maximum levels in samples 12 (190 °C) and 13 (210 °C) where sample 12 contained 15. 787 μg kg^−1^ and 16.652 μg kg^−1^ in sample 13. This is obviously well above the EC recommendation of 1 μgkg^−1^ for processed cereal-based foods and baby foods for infants and young children. Since malt can be used in foods intended for children (in bakery goods, bread as a colouring agent or diastatic activity supplement) [39], the results of this research are concerning, even more so since roasted, dark or black malts are used as colouring agents for cereal products. Malt flour is mostly used in bread, biscuits, crackers, crisp bread, breakfast cereals, infant foods, malted food drinks, sauces, sugar confectionary and vinegar production [39]. Most abundant light PAH was anthracene with values > 10 μg kg^−1^ for barley and >15 μg kg^−1^ for malt sample 1. Values in sample 10 are maximal values for this PAH and reach 31 μg kg^−1^. However, in sample 11 anthracene can no longer be detected, meaning that temperatures above 150 °C affect this compound to the point when it is no longer detectable. This could be due to chemical reactions between food ingredients or decomposition of anthracene at higher temperatures. This should be explored thoroughly.

These results differ from the ones that Mastanjević et al. [2] obtained in their research on commercially available malts. Nevertheless, the obtained results in this research could explain why ∑PAH4 was significantly higher in pilsner and cara-120 malt than in amber. Namely, amber malt is usually exposed to higher temperatures (100–150 °C) than pilsner malt (up to 80 °C) or cara-120 (100 °C); therefore, it is expected that amber should contain more PAHs. Namely, pilsner malt contained higher levels of benz [a] anthracene and pyrene than amber. Similarly, cara-120 malt contained significantly higher levels of PAH4, mostly as a result of benz [a] anthracene levels. This could be explained by the exposure temperatures since amber malt is exposed to higher temperatures for only a short period of time. However, this research also addressed the question of kilning time effect on PAH content by exposing malt to high temperatures, 190 °C and 210 °C, for a prolonged time of 2 h (Table 4). The results show that chrysene concentration significantly increases at higher temperatures and amounts to 64.161 μgkg^−1^ in sample 15, exposed to 210 °C for 2 h, which is four-fold higher than in sample 13 exposed to 210 °C for 1 h. The results for samples 12 and 14 are similar when exposed to 190 °C for 1 or 2 h. Sample 14 appears to contain almost two-fold higher concentrations of chrysene than sample 12. ∑PAH4 is the same as chrysene concentration since this is the only detectable PAH that constitutes ∑PAH4 and was also most abundantly detected in all samples mentioned in this paragraph.

Since there is no similar research, it is hard to compare the results with any other data. Hutt et al. [40] measured PAHs in corn and wheat before and after drying. ∑PAH4 value for corn sample dried using a yellow-flame burner was significantly higher than in wet corn. Chrysene value was above 32 μg kg^−1^. Perhaps the closest to this research are the data on coffee or cocoa beans roasted at different temperatures [25,26,41,42]. However, they are exposed to high temperatures for a much shorter time (up to 20 min) and the concentrations of PAHs were significantly lower than in this research, so this makes comparison inefficient. Nevertheless, research by Abballe et al. [43], published in 2021, described PAH content in different fractions of cocoa beans (whole bean, shell, liquor, cocoa powder and cocoa butter). There were two groups, one that was subjected to smoking and the other that did not undergo a smoking procedure at 120° for 60 min. In comparison to this research, the results showed lower values for all fractions of smoked samples, but they were still over the recommended levels for cocoa [29]. Cocoa shell, in particular, contained significant amounts of each constituent in ∑PAH4 (chrysene, benz [a] anthracene, benzo[b]fluoranthene and benzo [a] pyrene) resulting in a massive 116 μg kg^−1^. This could mirror our research since malt goes into processing as a complete grain, meaning that PAH levels for roasted malt are also high. Viegas et al. [44] reported that about 35% of PAHs from roasted coffee ends up in coffee brew. Regarding PAh levels in beer, research conducted by dos Santos et al. [45] reported that the PAH4 levels in beer ranged from 2.61 to 22.0 μg L^−1^. According to dos Santos, taking into an account that beer density is 1.0 g mL^−1^, it can be calculated that the sum in the samples (2.61–22.12 μg kg^−1^) was above the established limit. This indicated a serious potential health threat for beer consumers. Similar research [46,47] was conducted with concerning results. It is yet to be determined how many PAHs can end up in beer or other products to which malt or malt flour is added.

## 4. Conclusions

The conducted experiment confirmed that significant amounts of PAHs can be found in malt, particularly in special malts (dark, roasted). However, the obtained data indicate that certain PAHs disappear at higher temperatures. For example, phenanthrene is no longer detected at 100 °C, while fluorene and anthracene are no longer detected at 170 °C. Time of kilning is of great significance and the results show that ∑4PAH and ∑16PAH values are much higher when kilning lasts for 2 h at 210 °C (64.16 μg kg^−1^ for PAH4 and 68.75 μg kg^−1^ for PAH16), unlike when kilning duration is 1 h at 210 °C (PAH4 16.67 μg kg^−1^ and PAH16 24.32 μg kg^−1^). Further, the temperature of kilning is significant since higher levels of PAH4 and PAH16 were noted for samples exposed to 210 °C than in samples exposed to 190 °C (16.67 μg kg^−1^ at 210 °C vs. 15.79 μg kg^−1^ for PAH4 at 190 °C). Future studies should aim to pinpoint how to reduce the number of PAHs in malt, since malt is used in many foods consumed by children (bread, cookies, bakery products, etc.).

## Figures and Tables

**Table 1:** General micromalting scheme of barley samples.

Day	Micromalting Step and Operating Conditions		Sample Number
	Barley		0
1st	Immersion steeping for 5 h at 14 °C;		
	Dry steeping for 19 h at 14 °C, relative air humidity 95 %.		
2nd	Immersion steeping for 4 h at 14 °C;		
	Dry steeping for 20 h at 14 °C, relative air humidity 95 %.		
3rd	Immersion steeping for 1 h at 14 °C, relative air humidity 95 %.		
3rd–6th	Germination was carried out according to the scheme: 96 h at 14 °C Relative air humidity in each step was 95 %		
7th	Kilning was performed for 19 h, according to standard procedures for pale malt, after last germination hour; additional temperatures were set following the standard procedure.	50 °C for 16 h	1
		60 °C for 1 h	2
		70 °C for 1 h	3
		80 °C for 1 h	4
		90 °C for 1 h	5
		100 °C for 1 h	6
		110 °C for 1 h	7
		120 °C for 1 h	8
		130 °C for 1 h	9
		150 °C for 1 h	10
		170 °C for 1 h	11
		190 °C for 1 h	12
		210 °C for 1 h	13
		190 °C for 2 h	14
		210 °C for 2 h	15
	Malt degermination; packing in paper bags and storage;		

**Table 2:** List of analysed PAHs and designated abbreviations and IARC [38] classification.

Polycyclic Aromatic Hydrocarbon (PAH)	Abbreviation	IARC Carcinogenic Group *
benzo [*a*] pyrene	BaP	1
dibenz [*a,h*] anthracene	DBahA	2A
naphthalene	Nap	2B
benz [*a*] anthracene	BaA	
chrysene	CHR	
benzo [*b*] fluoranthene	BbFA	
benzo [*k*] fluoranthene	BkFA	
indeno [*1,2,3-cd*] pyrene	IP	
acenaphthene	Ane	3
fluorene	Fln	
phenanthrene	Phen	
anthracene	Ant	
fluoranthene	Flt	
pyren	Pyr	
benzo [*ghi*] perylene	BghiP	
acenaphthylene	Ane	4

* (group 1—carcinogenic to humans; group 2A—probably carcinogenic to humans; group 2B—possibly carcinogenic to humans; group 3—not classifiable as to its carcinogenicity to humans; group 4 —probably not carcinogenic to humans).

**Table 3:** PAH values (μgkg^−1^) in barley and malt samples exposed to different temperatures for 1 h.

Sample by Temperature	Ane	Fln	Ant	Phen	Flt	BaA	Pyr	CHR	DBahA	∑PAH 16	∑PAH4
0	0.48 ^i^	2.19 ^h^	11.50 ^f^	3.41 ^f^	1.18 ^k^	0.27 ^g^	0.31 ^l^	1.12 ^m^	0.38 ^j^	20.85 ^hi^	1.39 ^m^
1	1.16 ^h^	2.95 ^g^	15.09 ^e^	4.42 ^e^	1.35 ^j^	0.30 ^f^	0.40 ^k^	1.40 ^l^	0.40 ^j^	27.48 ^g^	1.70 ^l^
2	1.30 ^g^	3.60 ^f^	21.75 ^d^	5.66 ^d^	1.53 ^i^	0.30 ^f^	0.51 ^j^	1.54 ^k^	0.45 ^i^	36.64 ^f^	1.84 ^k^
3	1.32 ^g^	4.09 ^e^	22.47 ^cd^	6.06 ^c^	1.86 ^h^	0.30 ^f^	1.17 ^i^	1.73 ^j^	0.46 ^i^	39.46 ^def^	2.03 ^j^
4	1.32 ^g^	4.14 ^e^	24.15 ^bcd^	6.52 ^b^	2.05 ^g^	0.33 ^e^	1.17 ^i^	1.86 ^i^	0.47 ^hi^	42.03 ^cde^	2.19 ^i^
5	1.44 ^f^	4.39 _d_	24.80 ^bcd^	7.69 ^a^	2.24 ^f^	0.34 ^e^	1.21 ^h^	1.90 ^hi^	0.50 ^h^	44.51 ^bc^	2.24 ^i^
6	1.79 ^e^	4.89 ^c^	25.48 ^bc^	<LOQ	2.25 ^f^	0.41 ^d^	1.21 ^h^	1.93 ^h^	0.54 ^h^	38.49 ^ef^	2.33 ^h^
7	1.96 ^d^	4.97 ^c^	25.54 ^bc^	<LOQ	2.26 ^f^	0.43 ^c^	1.27 ^g^	2.10 ^g^	0.58 ^f^	39.11 ^def^	2.54 ^g^
8	2.18 ^c^	5.53 ^b^	27.32 ^b^	<LOQ	2.34 ^e^	0.45 ^b^	1.37 ^f^	2.52 ^f^	0.59 ^f^	42.33 ^cd^	2.98 ^f^
9	2.22 ^c^	7.41 ^a^	31.65 ^a^	<LOQ	2.39 ^d^	0.59 ^a^	1.54 ^e^	2.72 ^e^	0.87 ^e^	49.40 ^a^	3.31 ^e^
10	2.67 ^b^	<LOQ	34.78 ^a^	<LOQ	2.52 ^c^	0.61 ^a^	1.56 ^d^	3.06 ^d^	1.00 ^d^	46.18 ^ab^	3.66 ^d^
11	2.90 ^a^	<LOQ	<LOQ	<LOQ	2.53 ^c^	<LOQ	1.64 ^c^	9.99 ^c^	1.07 ^c^	18.15 ^i^	9.99 ^c^
12	2.95 ^a^	<LOQ	<LOQ	<LOQ	2.62 ^b^	<LOQ	2.35 ^b^	15.77 ^b^	1.39 ^b^	25.08 ^g^	15.79 ^b^
13	<LOQ	<LOQ	<LOQ	<LOQ	3.33 ^a^	<LOQ	2.71 ^a^	16.67 ^a^	1.59 ^a^	24.32 ^gh^	16.67 ^a^

^a^*^–^*^m^ Means are a result of triplicate within rows with different superscripts are significantly different (*p* < 0.05); <LOQ—below limit of quantification. Nap, Anl, BbFA, BkFA, BaP, BghiP, IP were below limit of detection.

**Table 4:** PAH values (μg kg^−1^) in samples exposed to 190 °C and 210 °C in dependence of exposure time.

Sample by Time vs. Temperature	Ane	Flt	Pyr	CHR	DBahA	∑PAH 16	∑PAH4
12/190 °C for 1 h	2.95 ^a^	2.62 ^c^	2.35 ^c^	15.77 ^c^	1.39 ^c^	25.08 ^c^	15.79 ^d^
13/210 °C for 1 h	<LOQ	3.33 ^b^	2.71 ^d^	16.67 ^d^	1.59 ^b^	24.32 ^d^	16.67 ^c^
14/190 °C for 2 h	<LOQ	3.79 ^a^	3.49 ^b^	28.77 ^b^	2.05 ^a^	38.11 ^b^	28.77 ^b^
15/210 °C for 2 h	<LOQ	<LOQ	4.59 ^a^	64.16 ^a^	<LOQ	68.75 ^a^	64.16 ^a^

^a–d^ Means within rows with different superscripts are significantly different (*p* <0.05); <LOQ—below limit of quantification. Nap, Anl, Fln, Ant Phen, BaA, BbFA, BkFA, BaP, BghiP, IP were below limit of detection.

## Data Availability

Data is contained within the article or supplementary material.

## Supplementary Materials

The following supporting information can be downloaded at: https://www.mdpi.com/article/10.3390/foods12030454/s1, Table S1: The average values for precision, reproducibility, accuracy, linearity, LOQ and LOD for PAH method validation.
